# Investigation of thermoelectric properties of flexible Ti_3_C_2_T_*x*_ MXene membranes

**DOI:** 10.1039/d5ra07980b

**Published:** 2026-01-16

**Authors:** Awais Irfan, Sajid Butt, Muhammad Faizan Masoud, Syed Rizwan, Muhammad Atif, Muhammad Abdul Basit

**Affiliations:** a Condensed Matter Physics Lab (CMP), Department of Space Science, Institute of Space Technology Islamabad 44000 Pakistan sajid.butt@ist.edu.pk; b Advanced Two-dimensional Materials & Devices Lab, Department of Physics & Astronomy, School of Natural Sciences, National University of Sciences and Technology (NUST) Islamabad 44000 Pakistan; c Department of Physics, Air University Islamabad Pakistan; d Department of Materials Science and Engineering, Institute of Space Technology Islamabad 44000 Pakistan

## Abstract

Two-dimensional MXenes have recently emerged as potential candidates for their excellent electrical and mechanical properties. We report the controlled modulation of thermoelectric properties in Ti_3_C_2_T_*x*_ flexible membranes *via* vacuum annealing. The as-prepared flexible membrane shows the highest electrical conductivity (∼5000 S m^−1^ at 373 K) and slightly estimated *ZT* value of 4.4 × 10^−3^ at 420 K due to preserved surface terminations and intercalated water contents. Notably, annealing at 300 °C enhances the Seebeck coefficient (∼450 µV K^−1^) and optimizes the power factor (∼105 µW m^−1^ K^−2^ at 450 K), whereas high temperature annealing (400 °C) significantly reduced thermoelectric performance due to excessive oxidation and degradation of the membrane. This work highlights that the tunability of MXene films through controlled annealing and surface functional group modification can significantly enhance the performance of thermoelectric materials for room- to mid-temperature range applications. The investigation of MXenes' thermoelectric properties opens new avenues for their use in flexible electronics and wearable devices.

## Abstract 2

Two-dimensional MXenes have recently emerged as potential candidates for their excellent electrical and mechanical properties.

## Graphical abstract

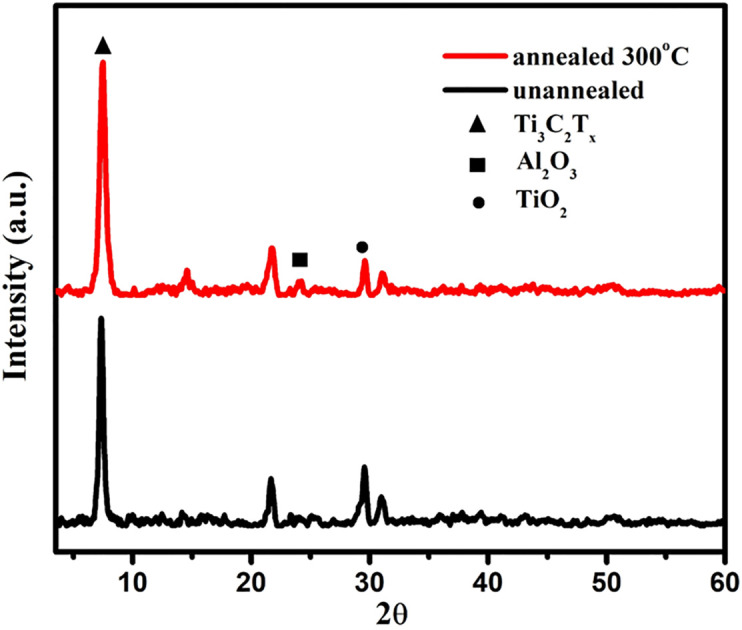

## Introduction

Demands for efficient and sustainable alternative energy solutions are rising due to the swift depletion of fossil fuel supplies and increased concerns about pollution and climate change.^1^ Thermoelectric materials can directly convert heat into electricity and have gained high attention in sustainable energy solutions.^1^ Since these solid-state devices do not have any moving components, they are highly reliable while being sufficiently compact to be used in a range of applications, from wearable electronics^2^ to industrial waste heat recovery.^3^ The dimensionless figure of merit 
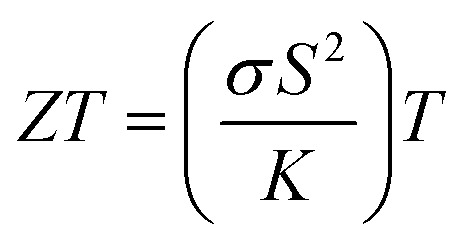
 determines the efficiency of a thermoelectric material where (*T*) is the absolute temperature, (*K*) is the thermal conductivity, (*S*) is the Seebeck coefficient, and (*σ*) is the electrical conductivity.^4^ The power factor (F) *σS*^2^, an important indicator of thermoelectric performance, is often used to evaluate thin films and flexible membranes, particularly when total thermal conductivity is not available.^5,6^ Recently, Copper selenide (Cu_2_Se) has gained much importance for improved thermoelectric performance in both bulk^7,8^ and thin films.^9,10^ On the other hand, Sb_2_Te_3_- and Bi_2_Te_3_-based alloys exhibit excellent thermoelectric properties due to their high Seebeck coefficient and low thermal conductivity near room temperature.^11–13^ However, despite their favorable thermoelectric properties, their mechanical characteristics pose challenges for integration into flexible devices.

MXenes, as an emerging class of 2D materials, have received immense importance due to their superior electrical, optical, and hydrophilic attributes as well as excellent chemical stability and mechanical flexibility.^14^ MXenes are represented by a formula of M_*n*+1_X_*n*_T_*x*_ (*n* = 1, 2, or 3) where M is an early transition metal (such as Sc, Ti, V, Mo, or Nb), X represents carbon or nitrogen, and T denotes surface terminal groups such as O, –OH, and –F. MXenes can be synthesized by selective etching that removes the X element (such as X = Al, Si, Ge, or Sn) from the parent carbide or nitride compounds.^15^ Since the initial discovery in 2011,^16^ there are about 30 different kinds of MXenes have been successfully synthesized, among which Ti_3_C_2_T_*x*_ remains the best studied and holds great prospects for energy storage,^17^ electromagnetic interference shielding,^18,19^ sensor,^20^ and catalysis^21^ all benefiting from the diverse merits in physical, chemical, and mechanical properties. The electrical conductivity of Ti_3_C_2_T_*x*_ MXene films is readily tunable. For instance, an exceptional (*σ*) value of approximately 200 S m^−1^^22^ has been achieved through high alignment and large-sized flakes prepared *via* blade-coating.^23^ In contrast, MXene films synthesized through HF etching exhibit a relatively lower *σ* value of approximately 15 S/m.^24^ Furthermore, the surface functional groups significantly influence the *σ* of Ti_3_C_2_T_*x*_ sheets,^25^ with -O-terminated Ti_3_C_2_T_*x*_ typically demonstrating higher conductivity than −F- or –OH-terminated counterparts. Additionally, theoretical studies showed that surface functionalization can cause significant modification in the electronic structure of MXenes, with certain M_2_C-type compositions (M = Sc, Ti, Zr, Hf, *etc.*) changing from metallic to semiconducting upon a –F, –OH, or –O termination. On the other hand, semiconducting MXenes such as Sc_2_C_2_, Ti_2_CO_2,_ and Zr_2_CO_2_ all have predicted moderate band gaps of (0.24–1.8 eV), have large Seebeck coefficients, especially at low temperatures, and therefore have a real opportunity for thermoelectric applications.

This study investigates the tailoring of thermoelectric properties of Ti_3_C_2_T_*x*_ MXene membranes through controlled annealing and by surface terminations, which can significantly enhance their thermoelectric performance.

### Experimentation

Ti_3_C_2_T_*x*_ MXene was synthesized by selectively etching the Al layer from the Ti_3_AlC_2_ MAX phase.^25,26^ Commercial-grade Ti_3_AlC_2_ powder was used as the starting material. The etching process involves slowly adding 1.0 g of sieved Ti_3_AlC_2_ powder, in small increments over 5–8 minutes, to a mixture of hydrofluoric acid (48 wt%, 1 mL), deionized water (3 mL), and hydrochloric acid (37 wt%, 6 mL), maintaining a volume ratio of 1 : 3 : 6. The reaction was carried out in a sealed Teflon-lined vessel under constant stirring at 450 rpm for 24 hours at 35 °C. Following etching, the suspension was repeatedly washed with deionized water until a neutral pH (≈7) was achieved. The etched material was collected by vacuum filtration and dried overnight at room temperature to obtain multilayer Ti_3_C_2_T_*x*_ MXene powder. Delamination was carried out using lithium chloride (LiCl, 99%) to obtain a stable colloidal suspension. The dried MXene powder was dispersed in 20 mL of deionized water, followed by the addition of 1 g of LiCl. The mixture was shaken for 8–10 minutes and stirred at 300 rpm for 24 hours. Subsequent centrifugation was performed at 3500 rpm for 5 minutes. This delamination step was repeated until a stable colloidal solution was achieved. The final sediment formed a viscous, clay-like paste, which was vacuum-filtered through Celgard membranes to produce freestanding MXene films. The thickness of the synthesized films was measured using a micrometer screw gauge and found to be approximately 21 µm. To further optimize their properties, the films were heat-treated at 100 °C and annealed at 400 °C for 1-hour under vacuum conditions.^26^

The crystal structure and phase purity were determined using X-ray diffraction (XRD) equipment made by ARL EQUINOX 3000 using equipped with Cu-Kα radiation. The surface chemical states of elements present were analyzed using X-ray photoelectron spectroscopy (XPS), of PHI QUANTERA SXM, having Al Kα radiation. The binding energies were calibrated with respect to the C 1s peak at ∼284.8 eV to account for surface charging effects. XPSPEAK41 software was used for the deconvolution of high-resolution spectra for the C 1s, Ti 2p, O 1s, and F 1 score levels. Scanning Electron Microscopy (SEM) MIRA3 TESCAN, coupled with an energy dispersive X-ray (EDX) detector, was utilized to analyze surface morphology and elemental composition. The electrical conductivity and Seebeck coefficient were measured utilizing the well-known four-probe method over the temperature range of 298–450 K by the Thermoelectric Parameter Test System (Joule Yacht-NAMICRO-3L).

## Results and discussion

These techniques collectively enabled a detailed understanding of structure–property linkage and its interdependence. The primary focus of this work is to correlate structural and electrical data to identify the dominant factors influencing charge transport, thereby contributing to the optimization of materials for thermoelectric applications.

**Fig. 1:**
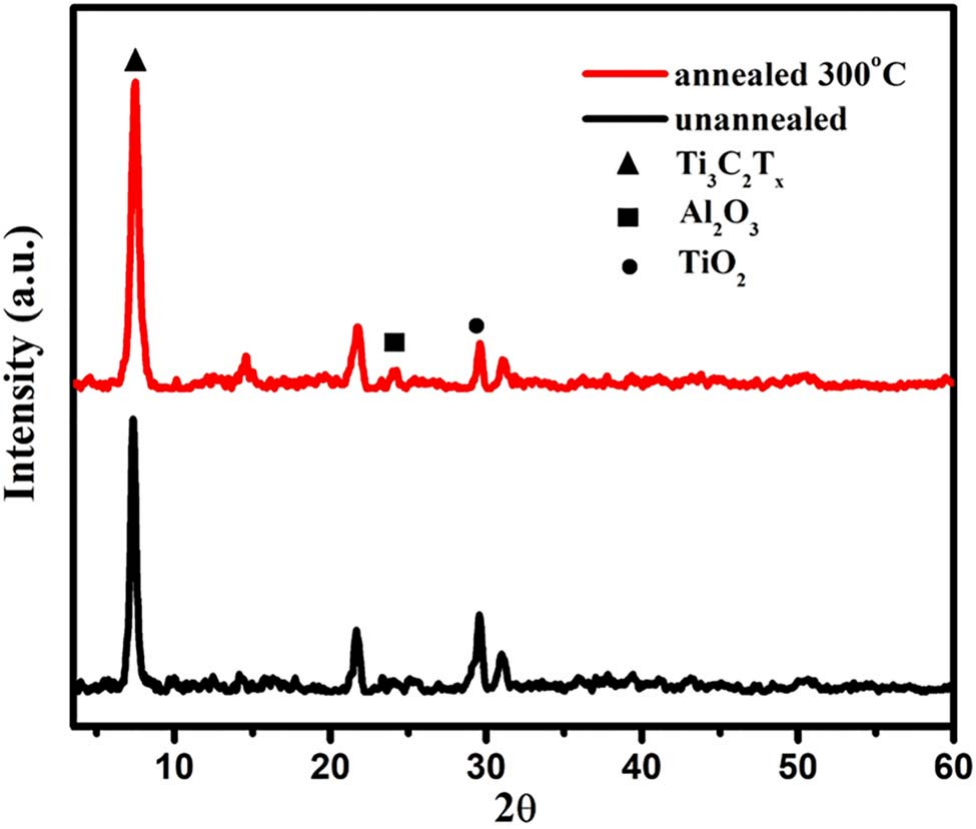
XRD pattern of Ti_3_C_2_T_*x*_ MXene flexible membranes un-annealed, annealed at 300 °C.

The structural changes in Ti_3_C_2_T_*x*_ MXene obtained from Ti_3_AlC_2_ through selective etching were established by XRD analysis, as shown in Fig. 1. The diffraction peaks of the samples are predominantly crystallized into sharp reflections from the hexagonal Ti_3_AlC_2_ MAX phase and can be indexed to the *P*6_3_/mmc space group, suggesting that the fabrication of the MXene-based free-standing flexible membrane was successfully achieved. Some weak peaks for Al_2_O_3_ also indicate some remaining aluminum oxide, referring to incomplete etching. The sample annealed at 300 °C shows a peak shift to higher 2*θ* values, which signifies the relatively reduced interlayer space, which is associated with the partial removal of intercalated water and surface functional groups –OH and –F, and correspondingly for the partial re-stacking of the MXene layers. Meanwhile, the presence of minor TiO_2_ phases based on partial oxidation of MXene surfaces upon heating is consistent with the previously reported data.^27^

**Fig. 2:**
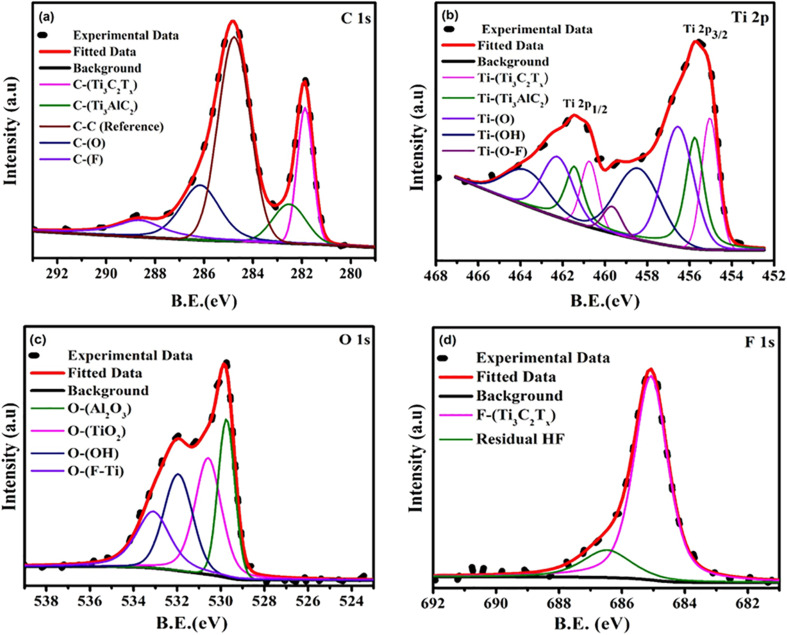
XPS analysis of Ti_3_C_2_T_*x*_ MXene flexible membranes (a) the high resolution spectra of C 1s from Ti_3_C_2_T_*x*,_ (b) the high resolution spectra of Ti 2p from Ti_3_C_2_T_*x*,_ (c) the high resolution spectra of O 1s from Ti_3_C_2_T_*x*,_ (d) the high resolution spectra of F 1s from Ti_3_C_2_T_*x*._

The high-resolution XPS spectra for C 1s, Ti 2p, O 1s, and F 1s were deconvoluted, as shown in Fig. 2. The C 1s spectrum, Fig. 2(a), shows a main peak at approximately 284.8 eV (C–C), used for calibration. Additional peaks for C–Ti_3_C_2_T_*x*_ and C–Ti_3_AlC_2_ indicate residual MAX phase. C–F and C–O peaks reflect surface terminations typical of HF etching, which influence electrical properties. The Ti 2p spectrum, as shown in Fig. 2(b), displays Ti–C, Ti–O, Ti–OH, and Ti–O–F contributions, with TiO_2_ peaks indicating surface oxidation in the synthesized sample. The XPS analysis confirms progressive oxidation of Ti^3+^ to Ti^4+^, accompanied by the loss of –F and –OH surface terminations and the growth of TiO_2_-like components within the membrane. Since TiO_2_ is electrically insulating, the increasing Ti^4+^ fraction introduces additional scattering centers and carrier-trapping sites, which collectively reduce carrier mobility and concentration. This behavior is fully consistent with the observed increase in activation energy and the corresponding decline in electrical conductivity.

A minor Ti_3_AlC_2_ signal confirms residual MAX phase, and the surface terminations are relevant to thermoelectric performance. The O 1s spectrum, as shown in Fig. 2(c), confirms TiO_2_ and OH–Ti formation in as as-synthesized sample. Signals from O–F–Ti and Al–O suggest etching by-products. These changes correlate with increased oxidation and reduced electrical conductivity on heat treatment. The F 1s spectrum shows peaks for F–Ti_3_C_2_T_*x*_ (∼684.8 eV) and residual HF (∼686 eV), as shown in Fig. 2(d). The reduced fluorine intensity indicates breakdown of Ti–F bonds and formation of insulating TiO_2_, contributing to conductivity loss.

**Fig. 3:**
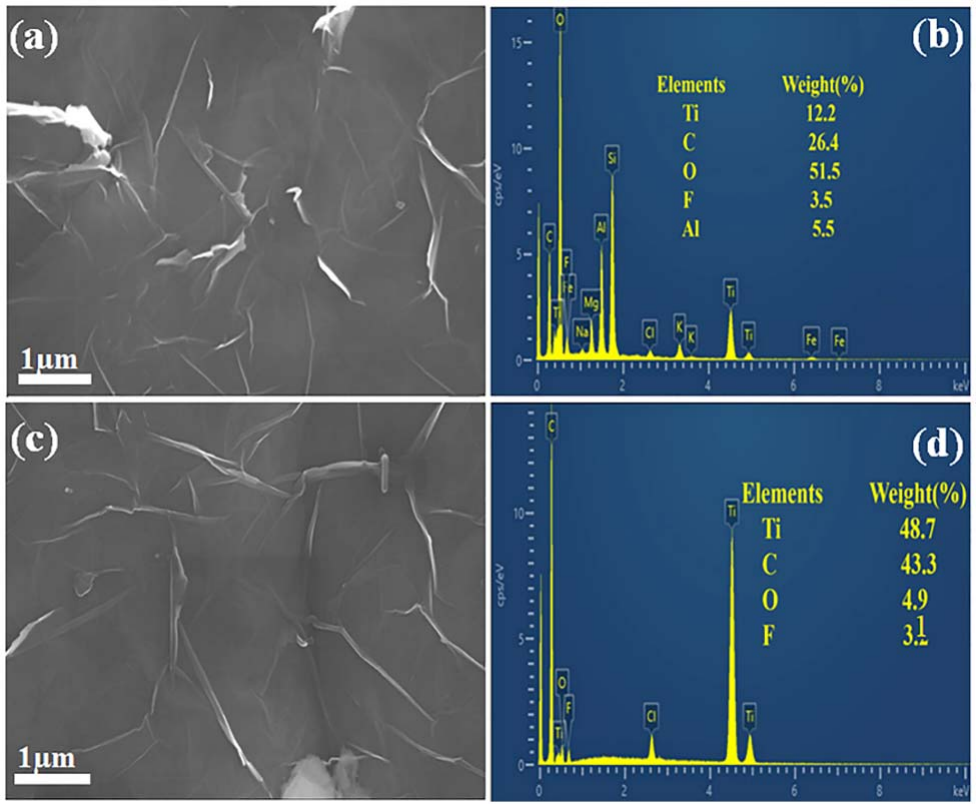
SEM and EDS analysis of Ti_3_C_2_T_*x*_ MXene flexible membranes (a) SEM of the un-annealed sample, (b) EDS of the un-annealed sample, (c) SEM of the sample annealed at 300 °C, (d) EDS of the sample annealed at 300 °C.

The morphological properties of unannealed and annealed Ti_3_C_2_T_*x*_ MXene membranes were investigated, as shown in Fig. 3. The SEM image of the un-annealed sample shows a well-exfoliated, layered morphology with network-like flakes characteristics of HF-etched Ti_3_C_2_T_*x*_ MXene, as shown in Fig. 3(a). The observed sheet-like morphology facilitates charge transport and enhances electrical conduction. The layered structure becomes denser and more aggregated, likely due to the removal of intercalated water molecules and partial decomposition of surface terminations upon vacuum annealing at 300 °C, as shown in Fig. 3(c). This restacking effect reduces interlayer spacing, thereby affecting charge transport properties. The elemental composition of Ti_3_C_2_T_*x*_ MXene membranes before and after annealing is shown in Fig. 3(b)–(d), respectively. The EDS spectrum of the un-annealed sample confirms the presence of titanium (Ti), carbon (C), and oxygen (O), along with trace amounts of fluorine (F) and aluminum (Al). Residual fluorine (F) and aluminum (Al) are likely remnants of the HF etching process, consistent with previous findings on Ti_3_C_2_T_*x*_ MXene synthesis.^28^ The elevated oxygen content is likely due to surface terminations (*e.g.*, –O, –OH) and adsorbed moisture, which promote hydrophilicity and exert a considerable impact on the material's electrical transport properties. Upon annealing at 300 °C, notable compositional changes were observed, as shown in Fig. 3(d). The Ti content increased, while the carbon level remained relatively constant. A pronounced reduction in F and Al signals indicates the breakdown of Ti–F surface terminations, accompanied by surface oxidation and the formation of TiO_2_-rich regions. This oxidation introduces insulating barriers that hinder charge carrier mobility, thereby reducing the electrical conductivity of the annealed MXene at elevated temperatures.

**Fig. 4:**
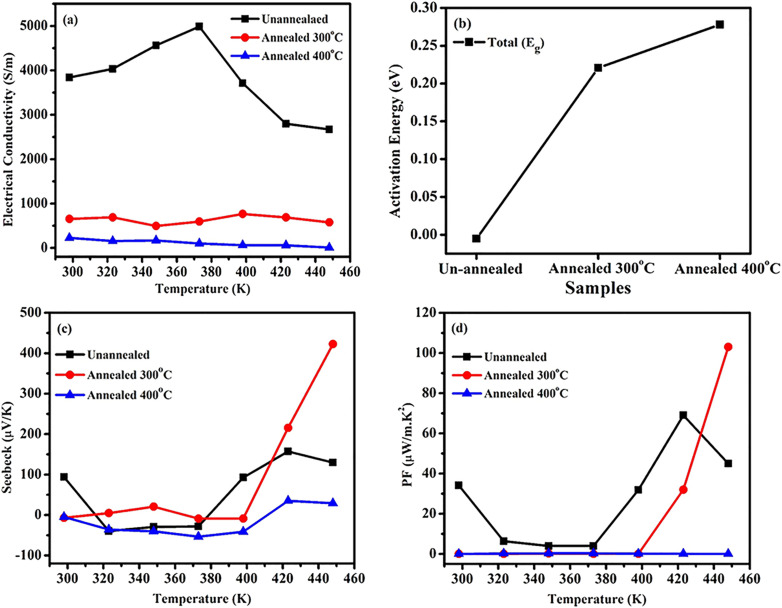
(a) Electrical conductivity (b) activation energy (*E*_a_) increases with annealing, indicating reduced carrier mobility. (c) Seebeck coefficient (*S*) peaks for the 300 °C annealed sample. (d) Power factor (PF).

Fig. 4(a) shows the temperature dependence of electrical conductivity for all the series un-annealed and annealed Ti_3_C_2_T_*x*_ MXene membranes over the range of 300 K to 460 K. At room temperature, the electrical conductivity of the un-annealed sample is approximately 4000 S m^−1^. As the temperature increases, the conductivity also rises, reaching a maximum value of 5000 S m^−1^ at 373 K. This increase, observed from 300 K to 373 K, is attributed to the removal of intercalated water molecules, which reduces interlayer spacing and enhances charge transport.^29^ However, above 373 K, the electrical conductivity begins to decline, likely due to structural degradation of MXene layers or the formation of an oxide phase such as TiO_2_, which exhibits lower conductivity. Oxidation at elevated temperatures leads to increased charge carrier scattering, further reducing conductivity. For the sample annealed at 300 °C, the room-temperature conductivity is about 700 S m^−1^, increasing slightly with temperature and reaching at approximately 900 S m^−1^ around 400 K. Beyond 400 K, the conductivity declines again, suggesting structural modifications or defect generation due to the annealing process. Excessive annealing temperatures can promote oxidation and structural degradation, further suppressing conductivity. This annealed sample exhibits semiconductor-like behavior across the measured temperature range.^30^ The 400 °C annealed sample displays the lowest electrical conductivity throughout the temperature range, with a room temperature value of approximately 300 S m^−1^. The pronounced reduction in conductivity at higher annealing temperatures is attributed to a combination of oxidation, alterations in surface terminations, and temperature-dependent defect dynamics.^31^

The activation energy for carriers was calculated using Arrhenius plots, as given by eqn (1).1
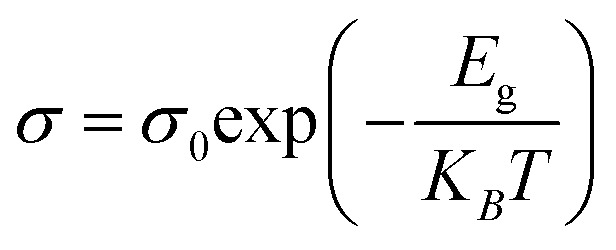
where *σ*_0_ is the temperature-independent part of the electrical conductivity, (*E*_g_) is the activation energy for conduction, *K*_*B*_ is the Boltzmann constant, and (*T*) is the absolute temperature. The activation energy calculated for the unannealed sample is negligible (≈0 eV), indicating metallic or semi-metallic transport behavior. Conversely, annealing at 300 °C and 400 °C under vacuum for 1 hour produced higher activation energies of approximately 0.22 eV and 0.28 eV, respectively, as shown in Fig. 4(b). This increase reflects a reduction in carrier concentration, consistent with removal of interlayer water, loss of electron-donating surface terminations (–OH, –F), and onset of oxidation. The higher *E*_g_ indicates that annealing shifts the transport from metallic toward thermally activated semiconducting behavior, consistent with the observed rise in Seebeck coefficient.

Fig. 4(c) presents the temperature-dependent Seebeck coefficient of Ti_3_C_2_T_*x*_. MXene membranes, illustrating the effect of annealing. The un-annealed sample exhibits n-type behavior (negative Seebeck coefficient) from 300 to 373 K, shifting to p-type above 373 K due to structural changes such as water desorption and oxidation-induced TiO_2_ formation, both of which reduce carrier concentration. At lower temperatures, electron-like carriers dominate due to the presence of electronegative surface terminations (–F, –OH) and intercalated water, which donate electrons or stabilize high electron density. Thus, the Seebeck coefficient is negative (n-type). Desorption of intercalated water, XRD peak shift confirmed. At ∼373 K, the combination of carrier depletion, Fermi-level lowering, and acceptor-state formation causes the Fermi level to move closer to the valence-band-like states of Ti_3_C_2_T_*x*_, as shown in eqn (2).^32^2
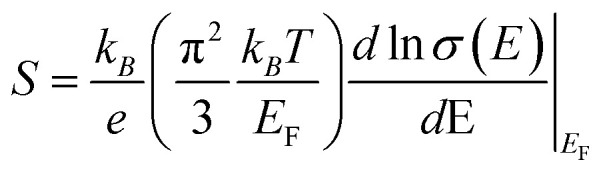
When the Fermi level approaches the valence band, the energy derivative term becomes positive, producing a positive Seebeck coefficient (p-type).

For the 300 °C annealed sample, the Seebeck coefficient remains low up to 400 K and then rises sharply to ∼450 µV K^−1^ at 450 K, The strong increase in Seebeck coefficient for the 300 °C annealed sample (∼450 µV K^−1^) is attributed to carrier depletion (higher *E*_a_, reduced terminations), formation of TiO_2_ restacking of MXene layers, These factors introduce an energy-filtering effect, where low-energy carriers are preferentially scattered while high-energy carriers contribute more strongly to transport. This inherently increases S even as *σ* decreases, a well-known mechanism in nanostructured thermoelectrics. Beyond this, it decreases, indicating further structural changes hindering carrier transport. The 400 °C annealed sample shows a stable, damped Seebeck response across the entire range. The absence of a sharp peak suggests increased oxidation and TiO_2_ content, which suppress charge mobility and degrade thermoelectric performance.^33^

The power factor (PF = *S*^2^*σ*) of all the series is shown in Fig. 4(d). The un-annealed sample shows a low PF value at lower temperatures, peaking at ∼75 µW m^−1^ K^−2^ around 430 K due to water desorption and an n-to-p-type transition that modifies carrier concentration and scattering. Above 430 K, it declines due to oxidation and increased carrier scattering. The 300 °C annealed sample exhibits a modest power factor up to 400 K and then rises sharply to ∼105 µW m^−1^ K^−2^ at 450 K, indicating optimized carrier concentration and reduced defects. Annealing processes can significantly influence the power factor of thermoelectric materials by altering their structural, electrical, and morphological properties.^34^ Beyond this, it drops rapidly, likely due to oxidation-induced degradation. In contrast, the 400 °C annealed sample maintains a low, stable power factor across all temperatures, suggesting excessive oxidation and phase transformation that hinder carrier transport. The formation of insulating TiO_2_ phases reduces carrier mobility and disrupts the Ti_3_C_2_T_*x*_ MXene network, degrading thermoelectric performance. Compared with recent MXene-based thermoelectric studies,^35^ as mentioned in Table 1 this work demonstrates notable advancements. The Ti_3_C_2_T_*x*_ film achieves a high Seebeck coefficient of ∼450 µV K^−1^ at ∼450 K among the highest reported for MXene systems at elevated temperatures. Unlike most studies limited to near-room-temperature measurements,^36^ our results extend into the mid-temperature regime, enhancing relevance for practical applications. The temperature-induced oxidation of MXene is shown to increase the Seebeck coefficient *via* carrier depletion and energy-filtering effects, while simultaneously modifying electrical conductivity, providing a mechanistic insight seldom addressed in prior literature. Additionally, the film maintains mechanical flexibility after thermal treatment, underscoring its potential for flexible thermoelectric devices. Collectively, these findings advance the development of high-performance and mechanically robust MXene-based thermoelectric materials.

**Table 1:** Comparison table summarizing thermoelectric properties from relevant reports

Literature review	T (K)	S (µvK^−1^)	*σ* (S m^−1^)	PF (µW mK^−2^)	*ZT*	Reference
Ti_3_C_2_T_*x*_ flexible membrane (300 °C annealed)	450	∼450	∼900	∼110	∼4.4 × 10^−3^ (420 K, USING *K*_*T*_ TOTAL LITERATURE range)	This work
MXene/Organics/TiS_2_ misfit flexible film	∼300	44.8	—	77.2	NOT REPORTED	40
Ti_3_C_2_T_*x*_ films (hydration & transport study)	300	−8.96	8200	VERY SMALL	NOT REPORTED	39
Neat Ti_3_C_2_T_*x*_ films (stacking-controlled, aligned MXene)	RT	UP TO ∼210	UP TO ∼20 000	UP TO ∼156	NOT REPORTED	41
Mo_2_TiC_2_T_*x*_/Nb_2_CT_*x*_ MXenes (representative TE reports & reviews)	300	Tens	VARIED	11–13	NOT REPORTED	42

### The electronic thermal conductivity (*κ*_*e*_) can be calculated by the Wiedemann–Franz law

**Fig. 5:**
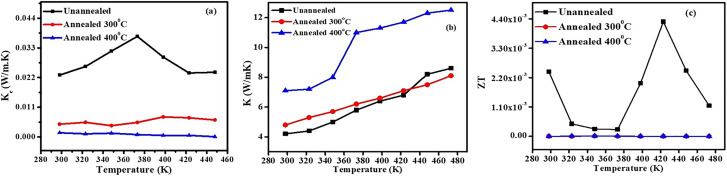
(a) The temperature dependent electronic thermal conductivity (*κ*_*e*_), (b) thermal conductivity values used for *ZT* estimation, adapted from ref. 39 with permission from the Royal Society of Chemistry, *J. Mater. Chem. C*, 2023, copyright 2023, and (c) the estimated *ZT* value, of all the series of samples.

*κ*_*e*_ *= L*_o_ *σT*, as shown in Fig. 5(a), where *L*_o_, *σ*, and *T* are the Lorentz number, electrical conductivity, and temperature, respectively. The un-annealed sample shows the highest electronic thermal conductivity (*κ*_*e*_), reaching 0.037 W m^−1^ K^−1^ near 380 K.^37^ Annealing at 300 °C decreases *κ*_*e*_, primarily due to the loss of surface terminations and water molecules, which contribute to electronic pathways and weaken charge transport. At 400 °C, severe oxidation leads to TiO_2_ formation,^38^ introducing charge traps and scattering centers that significantly obstruct electron transport, resulting in the lowest (*κ*_*e*_) in annealed samples. The measurement of total thermal conductivity, especially the lattice thermal conductivity (*κ*_*l*_) of flexible membranes, is challenging due to the unavailability of specialized equipment. Therefore, we have used approximated values of thermal conductivity (*κ*_*t*_) of unannealed and annealed MXene from reported data^39^ as shown in Fig. 5(b).

Fig. 5(c) shows the estimated *ZT* values as calculated by using approximated thermal conductivity. The un-annealed sample exhibits the highest *ZT*, reaching 4.4 × 10^−3^ near 420 K, due to its high electrical conductivity and moderate Seebeck coefficient, supported by intact surface terminations (–OH, –F, –O) and intercalated water that facilitate metallic-like transport. Its relatively low electronic thermal conductivity also contributes to an enhanced *ZT*. In contrast, the 300 °C-annealed sample shows a reduced and nearly constant *ZT* (∼0.05) across the temperature range, attributed to the partial loss of terminations and water, which lowers carrier concentration and conductivity. Although minor Seebeck enhancement may occur due to energy filtering at defect sites, it is outweighed by increased scattering and reduced transport. The 400 °C-annealed sample exhibits negligible *ZT* due to severe oxidation.

## Conclusion

This study shows that the thermoelectric performance of Ti_3_C_2_T_*x*_ MXene membranes can be tuned *via* controlled annealing. Un-annealed samples, rich in –O, –OH, –F terminations and interlayer water, exhibited the highest electrical conductivity (∼5000 S m^−1^ at 373 K) and estimated *ZT* value of 4.4 × 10^−3^ at 420 K. Annealing at 300 °C enhanced the Seebeck coefficient and achieved a peak power factor of ∼105 µW m^−1·^K^−2^ at 450 K due to optimized carrier scattering and partial surface group removal. This work demonstrates one of the highest high-temperature Seebeck coefficients, 450µV K^−1^ reported for MXene films, along with preserved flexibility and a clear oxidation-driven transport mechanism. However, annealing at 400 °C led to severe oxidation and TiO_2_ formation, reducing conductivity and thermoelectric performance. Overall, the findings highlight the importance of surface terminations and demonstrate that optimized annealing can improve performance while preserving film integrity. This offers a pathway for developing MXene-based flexible thermoelectric devices for low-temperature energy harvesting.

## Conflicts of interest

All the authors declare that there is no conflict of interest.

## Data Availability

Data will be provided upon request.
